# The role of lipid emulsions containing omega-3 fatty acids for medical and surgical critical care patients

**DOI:** 10.1186/s13054-024-05053-4

**Published:** 2024-08-12

**Authors:** Christian Stoppe, Robert G. Martindale, Stanislaw Klek, Philip C. Calder, Paul E. Wischmeyer, Jayshil J. Patel

**Affiliations:** 1 University Hospital Würzburg, Department of Anaesthesiology, Intensive Care, Emergency and Pain Medicine, Wuerzburg, Germany; 2 Department of Cardiac Anesthesiology and Intensive Care Medicine, German Heart Center Charité Berlin, Berlin, Germany; 3 Department of Surgery, Oregon Health and Science University, Portland, OR USA; 4 Surgical Oncology Clinic, The Maria Sklodowska-Curie National Cancer Institute, Krakow, Poland; 5 Faculty of Medicine, University of Southampton, Southampton, UK; 6 NIHR Southampton Biomedical Research Centre, University Hospital Southampton NHS Foundation Trust and University of Southampton, Southampton, UK; 7 Division of Anesthesiology and Critical Care Medicine, Duke University Hospital, Durham, NC USA; 8 Division of Pulmonary and Critical Care Medicine, Medical College of Wisconsin, Milwaukee, WI USA

**Keywords:** Omega 3, Parenteral nutrition, Lipids, Critical illness, Guidelines, Consensus

## Abstract

In critical illness the regulation of inflammation and oxidative stress can improve patient outcomes, and thus omega-3 polyunsaturated fatty acids (PUFAs) have been used as part of parenteral nutrition (PN) owing to their potential anti-inflammatory effects. The international lipids in PN Summit, encompassed discussions and the production of consensus guidelines concerning PN intravenous lipid emulsion (ILE) use in critical care. The Lipid Summit participants agreed that the inclusion of fish oil in ILEs is associated with meaningful clinical benefits without signals of harm, based on a strong biological rationale and current clinical evidence. Decisions concerning ILE choice should be made based on current evidence, thus addressing clinical requirements for guidance, particularly as further definitive evidence seems unlikely to occur. In addition, a future of individualized ICU care is envisioned, yielding better clinical outcomes. This approach will require the greater use of intelligent study designs incorporating the use of biomarkers of omega-3 derivatives, inflammatory-resolving processes, and/or muscle protein breakdown.

## Introduction

Clinical outcomes during critical illness are influenced by the balance between inflammation/oxidative stress and the anti-inflammatory immune response [1]. Normally, antioxidant and anti-inflammatory defense systems regulate inflammation and oxidative stress, but this ability can be compromised during critical illness, exacerbated by advanced age, comorbidities, and illness severity [2]. Omega-3 polyunsaturated fatty acids (PUFAs) used in parenteral nutrition (PN) have been investigated in research and clinical practice, owing to their potential anti-inflammatory attributes [3].

The purpose of this commentary is to: (1) provide a short overview of the biological role of omega-3-PUFAs in critical illness; (2) summarize related clinical evidence; (3) discuss potential controversies in this area; and (4) outline guidance and future directions. The impetus for this commentary is derived from the international Lipids in Parenteral Nutrition Summit, held on November 3 and 4, 2022, in New Orleans, USA, involving a panel of international experts with clinical and scientific experience of PN to discuss biological and clinical aspects of lipids used in PN [4]. Consensus statements were produced to provide practical guidance regarding the use of lipid emulsions in PN that complement societal critical care nutrition guidelines [5–7], a full set of which will be published elsewhere [4]. Table 1 shows a subset of the consensus statements which are relevant to the critical care setting.

**Table 1:** Consensus statements from the lipids in PN Summit relevant to medical and surgical ICU patients

Consensus statement	Voting
6. In adult medical and surgical ICU patients requiring PN, ILEs are an integral part of PN	Agree: 18 (100%) Don’t agree: 0 Don’t wish to answer: 0
7. The use of ILEs containing fish oil should be considered during the first week of ICU admission in adult patients requiring PN including surgical and critically ill patients, based on biological plausibility and associated clinical benefits	Agree: 18 (100%) Don’t agree: 0 Don’t wish to answer: 0
8. There is accumulating scientific evidence from clinical trials, systematic reviews, and meta-analyses to demonstrate that ILEs containing fish oil have clinically meaningful advantages over ILEs without fish oil when used in adult patients requiring PN including surgical and critically ill patients with a favorable risk–benefit ratio. Furthermore, the use of ILEs containing fish oil as part of PN has shown to be cost-effective in these populations	Agree: 17 (94.4%) Don’t agree: 0 Don’t wish to answer: 1 (5.6%)
9. In adult medical and surgical ICU patients, the total lipid dose is in general up to 1.5 g lipids/kg/day (including non-nutritive lipid sources such as propofol). A minimum dose of ILE should be given to prevent EFA deficiency	Agree: 17 (94.4%) Don’t agree: 1 (5.6%) Don’t wish to answer: 0
10. Based on currently available clinical data, we recommend 0.1–0.2 g fish oil/kg/day, provided by lipid emulsions containing fish oil, for adult medical and surgical ICU patients requiring PN	Agree: 17 (94.4%) Don’t agree: 0 Don’t wish to answer: 1 (5.6%)
11. The use of mixed lipid emulsions containing soybean oil, olive oil, MCTs, and/or fish oil, at the recommended dose, has not been shown to lead to EFAD in clinical practice. A 100% fish oil ILE has also not been shown to lead to EFAD in clinical practice	Agree: 18 (100%) Don’t agree: 0 Don’t wish to answer: 0
12. The concentrations of triglycerides (TGs) in serum should be within local or regional guidelines in adults during infusion. If the level is high, ensure the blood sample was drawn from an appropriate location. If the level exceeds for example 400 mg/dL (4.5 mmol/L) investigate secondary causes. Serum TGs should be assessed prior to the beginning of infusion in all patients	Agree: 17 (100%) Don’t agree: 0 Don’t wish to answer: 0
13. When mechanical circulatory support systems (e.g. extracorporeal membrane oxygenation; ECMO) or the use of cardiopulmonary bypass are required, the function of the membrane oxygenator should be closely overseen during lipid emulsion administration to monitor for the potential risk of clotting, which has occurred in very few cases. Additionally, when calculating the lipid intake, non-nutritional lipid sources (e.g. propofol) should be taken into account and closely monitor TG levels. Lipid emulsions may be administered as a continuous infusion over 12–24 h through a remote central venous line (prevent bolus application and infusion directly into the ECMO circuit)	Agree: 15 (83.3%) Don’t agree: 0 Don’t wish to answer: 3 (16.7%)
32. Supplemental PN is a combination of oral/EN and PN. It may be considered as a strategy with the intent to increase macronutrient delivery and to maintain/improve the nutritional status of patients such as critically ill (acute phase), surgical, and cancer patients if oral or EN is insufficient. ILEs are an integral part of supplemental PN	Agree: 17 (100%) Don’t agree: 0 Don’t wish to answer: 0
33. Administration of supplemental PN through a peripheral line can be considered over a short period of time when central line access is unavailable or as a bridge until central line access is available. ILEs are an integral part of peripheral PN	Agree: 17 (94.4%) Don’t agree: 1 (5.6%) Don’t wish to answer: 0
39. Nutrition societies should issue guidelines and recommendations addressing clinical validity when performing a systematic review. Differences in inclusion/exclusion criteria and methodology can result in significant differences in outcomes and conclusions. Translation of systematic review conclusions into clinical guidelines is also affected by many factors including the intent of the convening body, geographical regulations impacting clinical options, and balance between clinical requirement and need for additional definitive evidence	Agree: 18 (100%) Don’t agree: 0 Don’t wish to answer: 0

These consensus statements were formulated and voted on by the expert panel (listed below) participating in the International Lipids in PN Summit 2022, and thus represent the collective opinion of the summit experts informed by scientific evidence. The consensus summit experts who attended this meeting were: Magnus Bäck, Sweden; Philip Calder, UK; Sarah Cogle, USA; Valerio Chiurchiù, Italy; David Evans, USA; Leah Gramlich, Canada; Martin Hersberger, Switzerland; Stanislaw Klek, Poland; Robert Martindale, USA; Stephen McClave, USA; Bettina Mittendorfer, USA; Manpreet Mundi, USA; Maurizio Muscaritoli, Italy; Reid Nishikawa, USA; Jayshil Patel, USA; Lorenzo Pradelli, Italy; Martin Rosenthal, USA; Charles Serhan, USA; Christian Stoppe, Germany; Kelly Tappenden, USA; Dan Waitzberg, Brazil; Malissa Warren, USA; Paul Wischmeyer, USA. (Note not all experts were present for all sessions of the meeting/voted on all of the consensus statements.)

ECMO, extracorporeal membrane oxygenation; EFA: essential fatty acid; EFAD, essential fatty acid deficiency; EN, enteral nutrition; ICU, intensive care unit; ILE, intravenous lipid emulsion; MCT, medium-chain triglyceride; PN, parenteral nutrition; TG, triglyceride

## Omega-3 polyunsaturated fatty acids: biologic aspects

**Fig. 1:**
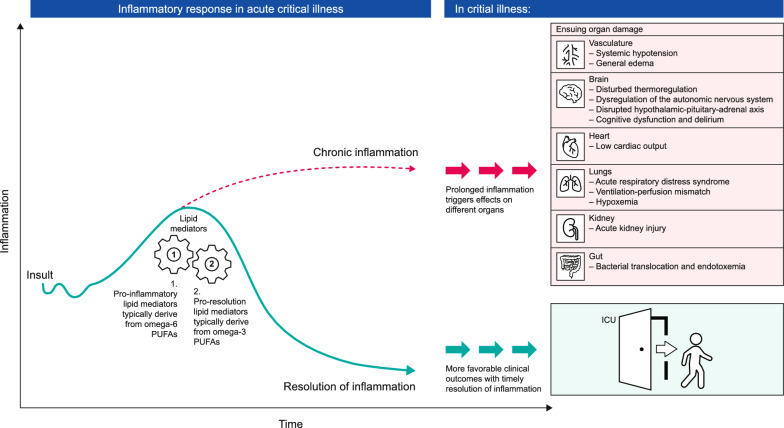
The acute inflammatory response to critical illness and its resolution or progression to chronic inflammation. Pro-resolution lipid mediators (i.e. resolvins, protectins, and maresins) typically derive from omega-3 polyunsaturated fatty acids (PUFAs). Pro-inflammatory lipid mediators (i.e. prostaglandins or leukotrienes) typically derive from omega-6 PUFAs. In critical illness, prolonged inflammation triggers effects on different organs that can result in organ damage. However, the timely resolution of inflammation is associated with more favorable clinical outcomes [1, 2, 8–10]

Excessive and uncontrolled inflammation is a hallmark of critical illness, and the ability of the host response to resolve inflammation and return to homeostasis has implications on clinical outcomes [1]. Resolution of inflammation is now understood to be a highly coordinated and biosynthetically active programmed response in which specialized pro-resolving mediators (SPMs) take on important coordinating tasks [8]. Arachidonic acid and long-chain omega-3 PUFAs (i.e. eicosapentaenoic acid [EPA] and docosahexaenoic acid [DHA]) are precursors for SPMs. SPM ‘families’ include lipoxins (from arachidonic acid) and resolvins, protectins, and maresins (from long-chain omega-3 PUFAs). Each SPM family has distinct roles in inflammation resolution—actively disrupting inflammatory pathways and shifting the immune response towards resolution and homeostasis (Fig. 1) [1, 2, 8–10]. Contrary to many common anti-inflammatory drugs, immune modulation and tissue repair involving SPMs occurs without compromising host defences [8, 9].

In addition to their inflammation-resolving effects [3], omega-3 PUFAs potentially counteract loss of muscle mass [11]. Preclinical investigations suggest that increased incorporation of EPA and DHA into membrane phospholipids leads to enhanced muscle protein synthesis and/or decreases the expression of factors regulating muscle protein breakdown [11]. The extent of these effects, however, may further depend on factors such as protein intake and age. Current clinical evidence is predominantly from oral supplementation in ageing populations and/or chronic diseases [9], rather than critically ill patients.

Lipids are an integral part of PN in all settings where PN is required, and soybean oil has traditionally been used as the intravenous lipid emulsion (ILE) of choice for PN. The 7:1 omega-6 to omega-3 PUFA ratio of soybean oil is important, since most lipid mediators derived from omega-6 PUFAs are pro-inflammatory [3]. In recent years, mixed-oil ILEs have been formulated to reduce their omega-6 PUFA content, partially replacing soybean oil with medium-chain triglycerides (MCTs), olive oil, and/or fish oil (which is rich in EPA and DHA) [3–5, 12].

## Evidence for benefits of PN containing omega-3 PUFAs in critically ill patients

Numerous clinical studies have evaluated mixed-oil ILEs (with or without fish oil) in different critically ill patient populations, with meta-analyses providing supplementary information [7, 12–14]. Results of most systemic reviews/meta-analyses indicate that ILEs containing fish oil have beneficial effects on the inflammatory response and clinical outcomes [12–14], with a notable exception [7]. One meta-analysis screened 1054 studies, including 26 randomized controlled trials (RCTs) and 1733 patients, finding that PN formulations containing less omega-6 PUFAs (any mixed-oil ILE) were associated with a significant decrease in hospital length of stay (LOS), and a trend towards reduction in 28-day mortality, ICU LOS, and shorter duration of mechanical ventilation [12]. Among the different mixed-oil ILEs, PN containing fish oil reduced the duration of ICU care and the rate nosocomial infection, and a trend towards shorter hospital LOS was observed [12]. These results aligned with a previous large meta-analysis, including 24 RCTs and1421 patients, that reported fewer infections, fewer cases of sepsis, and shorter durations of both hospital and ICU LOS [13]. A subsequent network-meta-analysis by the same group compared PN with and without fish oil (any mixed-oil ILE without fish oil), confirming these findings [14]. Notz et al. also reported benefits for fish oil on mortality rates compared with placebo when fish oil was used as a stand-alone ILE [12]—though the use of pure fish-oil ILEs for adults is experimental and should not (yet) be applied outside a formal clinical study setting.

The recent international Lipid Summit (Table 1) [4], the European Society for Clinical Nutrition and Metabolism (ESPEN) [5], and the Canadian Critical Care Nutrition Organization [6], have all acknowledged the advantages of PN containing fish oil (compared with PN without fish oil) for critically ill patients. In contrast, the 2022 American Society of Parenteral and Enteral Nutrition (ASPEN) critical care nutrition guideline update provided a weak recommendation for short-term use of either mixed-oil or pure soybean-oil ILEs in critically ill patients [7]. The meta-analyses informing this recommendation included only seven studies of mixed ICU patients [7] compared with 26 and 24 studies included in other recent meta-analyses [12, 13]. These ASPEN guidelines resulted in scientific discussions in which some co-authors of this commentary were involved [15–17]. For future related assessments and subsequent recommendations, we consider it important to put applied approaches and findings into a broader context by, for example, discussing results in comparison with the literature, or by formulating more specific recommendations regarding clinicians’ choice of lipid emulsion.

## Challenges of data generation related to omega-3 PUFA studies in critical illness

Studies evaluating the relationship between health outcomes and lipids used in PN are often inconclusive, but this also applies for any potential link between health and nutrition [18]. There are multiple explanations for such observations. RCTs evaluating omega-3 PUFAs in critically ill patients tend to have methodological shortcomings (as do many studies involving critically ill patients), resulting in an overall lower-grade of evidence compared with other studies such as those testing therapeutic safety and efficacy [4, 18]. Comparison of complex PN formulations, variable length of PN administration, and comparatively low patient numbers can contribute to difficulties in interpreting RCT data in this field. Furthermore, critical illness is a heterogeneous state, and thus patients may respond differently to the same interventions [19]. In addition, critical care nutrition trials per se are subject to biases, which are difficult or impossible to reconcile [18]. Such effects include (beside sample heterogeneity) a suboptimal understanding of the biological mechanisms and unknown modifiers of treatment effects by nutrition. For example, the complex metabolic response to a stress situation may induce uncontrolled catabolism and the development of resistance to anabolic signals [20]. Likewise, the extensive interplay between macronutrient dose, timing, and route of administration in critical care nutrition is poorly understood [21]. In forthcoming investigations some of these challenges can be overcome by optimization of trial designs and/or the enhanced use of biomarkers to help define nutritional risk, metabolic heterogeneity, and/or when an individual may respond to a nutrition intervention [21]. Until such data are available perhaps the existing body of evidence should be considered sufficient to provide guidance for clinical care.

## Guidance and future directions for the use of omega-3 PUFAs in critically ill patients: await definitive evidence, or take decisions based on available information?

The Lipid Summit participants advocate a balanced approach: providing clinicians with guidance based on preclinical and clinical evidence to help choose nutritional interventions in critically ill patients—but simultaneously acknowledging the desirability of additional, definitive evidence regarding complex subjects in clinical nutrition (Table 1, Statement 39, 100% agreement). Regarding choice of ILE for critically ill patients requiring PN, the Lipid Summit participants agreed, based on a strong biological rationale and current existing clinical evidence (as outlined above), that the inclusion of fish oil in ILEs is associated with meaningful clinical benefits without signals of harm (Table 1, statement 8; 94% agreement).

However, should we continue to work towards definitive evidence for the efficacy of ILE formulations in critically ill patients (e.g. by conducting further larger/higher-quality studies on ILEs in PN for ICU patients), or should we allocate resources towards a better understanding of patient identification, metabolic heterogeneity, and guided responses to nutrition intervention(s)? The former is a scenario that most of us consider somewhat unrealistic. We recommend the latter approach, leading towards more individualized treatment guided by biomarkers and based on multidimensional assessments. As critically ill patients are a heterogenous group, concepts have been recently proposed that promote individualizing patient care (known as a ‘treatable treat’) [19]. Translating this promising approach from theory into practice by following evidence-based medicine principles will require intelligent study designs with biomarkers at the core. Indicators of inflammatory-resolving processes and/or muscle protein breakdown will be essential, including markers derived from omega-3 PUFAs.

## Conclusion

The Lipid Summit participants envision a future of individualized ICU care and are confident that designing studies rooted in precision medicine will promote such care, yielding better clinical outcomes. Furthermore, we should be able to apply our knowledge concerning ILEs containing DHA and EPA, and their SPM derivatives, to produce more informative randomized controlled trial protocols and collection of appropriate data to produce more definitive clinical recommendations.

## Data Availability

No datasets were generated or analysed during the current study.

## Abbreviations

ASPEN: American Society of Parenteral and Enteral Nutrition
DHA: Docosahexaenoic acid
ECMO: Extracorporeal membrane oxygenation
EFA: Essential fatty acid
EFAD: Essential fatty acid deficiency
EN: Enteral nutrition
EPA: Eicosapentaenoic acid
ESPEN: European Society for Clinical Nutrition and Metabolism
ICU: Intensive care unit
ILE: Intravenous lipid emulsion
LOS: Length of stay
MCT: Medium-chain triglyceride
PN: Parenteral nutrition
PUFA: Polyunsaturated fatty acid
RCT: Randomized controlled trial
SPMs: Specialized pro-resolving mediators
TG: Triglyceride
